# High parasite diversity in the amphipod *Gammarus lacustris* in a subarctic lake

**DOI:** 10.1002/ece3.6869

**Published:** 2020-10-05

**Authors:** Jenny C. Shaw, Eirik H. Henriksen, Rune Knudsen, Jesper A. Kuhn, Armand M. Kuris, Kevin D. Lafferty, Anna Siwertsson, Miroslava Soldánová, Per‐Arne Amundsen

**Affiliations:** ^1^ Marine Science Institute University of California Santa Barbara Santa Barbara CA USA; ^2^ Department of Arctic and Marine Biology Faculty of Biosciences, Fisheries and Economics UiT The Arctic University of Norway Tromsø Norway; ^3^ Department of Ecology, Evolution and Marine Biology University of California Santa Barbara CA USA; ^4^ Western Ecological Research U.S. Geological Survey Santa Barbara CA USA; ^5^ Institute of Marine Research Ecosystem Processes Research Group Tromsø Norway; ^6^ Institute of Parasitology, Biology Centre Czech Academy of Sciences České Budějovice Czech Republic

**Keywords:** Amphipod, Cestoda, food web, Trematoda, trophically transmitted parasites

## Abstract

Amphipods are often key species in aquatic food webs due to their functional roles in the ecosystem and as intermediate hosts for trophically transmitted parasites. Amphipods can also host many parasite species, yet few studies address the entire parasite community of a gammarid population, precluding a more dynamic understanding of the food web. We set out to identify and quantify the parasite community of *Gammarus lacustris* to understand the contributions of the amphipod and its parasites to the Takvatn food web. We identified seven parasite taxa: a direct life cycle gregarine, *Rotundula* sp., and larval stages of two digenean trematode genera, two cestodes, one nematode, and one acanthocephalan. The larval parasites use either birds or fishes as final hosts. Bird parasites predominated, with trematode *Plagiorchis* sp. having the highest prevalence (69%) and mean abundance (2.7). Fish parasites were also common, including trematodes *Crepidostomum* spp., nematode *Cystidicola farionis*, and cestode *Cyathocephalus truncatus* (prevalences 13, 6, and 3%, respectively). Five parasites depend entirely on *G. lacustris* to complete their life cycle. At least 11.4% of the overall parasite diversity in the lake was dependent on *G. lacustris*, and 16% of the helminth diversity required or used the amphipod in their life cycles. These dependencies reveal that in addition to being a key prey item in subarctic lakes, *G. lacustris* is also an important host for maintaining parasite diversity in such ecosystems.

## Graphical abstract

We set out to identify and quantify the parasite community of *Gammarus lacustris* to understand the contributions of the amphipod and its parasites to the Takvatn food web. We identified seven parasite taxa: a direct life cycle gregarine, *Rotundula* sp., and larval stages of two digenean trematode genera, two cestodes, one nematode, and one acanthocephalan. We found that *G. lacustris* is not only a key prey item in subarctic lakes, it is also an important host for maintaining parasite diversity in such ecosystems.

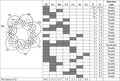

## 1 INTRODUCTION

There is often more than meets the eye when examining food web ecology. Network concepts such as connectance (Lafferty et al., 2006) and robustness (Lafferty & Kuris, 2009) have been well described for several food webs, including webs that consider infectious agents. Here, we look at a key species in the consumer dynamics of the Norwegian subarctic lake Takvatn (hereafter “Takvatn”; “vatn” means “lake” in Norwegian), the amphipod *Gammarus lacustris*, and reveal that it plays a central role in parasite transmission to the main predators in the lake, including several birds and three abundant fish species.

Amphipods of the genus *Gammarus* are common across a diverse range of lentic and lotic habitats in the northern hemisphere (Bousfield, 1958; Karaman & Pinkster, 1977; Väinölä et al., 2008). *Gammarus* species are omnivorous and function mainly as shredders, processing large organic matter (detritus) into smaller pieces that are consumed by other macroinvertebrates (Kelly et al., 2002; MacNeil et al., 1997). Gammarids are further significant prey items for fishes and are also eaten by birds and other vertebrates and invertebrates (reviewed in MacNeil et al., 1999), making them a highly connected species and thus a hub in the food web. Taken altogether, *Gammarus* spp. play an important role in aquatic ecosystems by contributing substantial biomass and through their roles as predator and prey (Kelly et al., 2002; MacNeil et al., 1997, 1999).

*Gammarus* spp. are often intermediate hosts for diverse parasites (Bojko & Ovcharenko, 2019; Denny, 1969), which is likely due to their high connectance in food webs and functional roles in ecosystems (based on Locke et al., 2014). Parasites can alter food web dynamics by making intermediate hosts more susceptible to predation by final hosts (Lafferty & Shaw, 2013; Thomas et al., 1999). For example, acanthocephalans, cestodes, and trematodes can alter the phenotype and/or behavior of their gammarid intermediate host, often with a result of increased predation by final host birds or fishes (Bakker et al., 1997; Helluy & Thomas, 2003; Hindsbo, 1972; Knudsen et al., 2001). Parasites also affect the feeding ecology of gammarids by decreasing their shredding activity (Dianne et al., 2014; Labaude et al., 2017; Medoc & Beisel, 2011). Hence, parasitism of amphipods can alter food web dynamics through bottom‐up (increased predation on these amphipods) and top‐down (altered feeding ecology) mechanisms. A single host is typically infected by multiple parasite species that form a dynamic assemblage (Holmes & Price, 1986; Poulin, 2014), and gammarids are no exception. Coinfections with two manipulative parasites further complicate parasite‐induced behavioral changes in gammarids (Cezilly et al., 2014; Haine et al., 2005). The ecosystem effects of parasitism may be particularly profound if the intermediate host is a key species or hub in the aquatic food web (Sures et al., 2017), and gammarids often play such a role in lacustrine ecosystems. To fully understand the ecological role of parasites at the ecosystem level in Takvatn, it is necessary to evaluate the entire parasite assemblage of a gammarid population.

*Gammarus lacustris* is one of the most widespread freshwater amphipod species, with a nearly circumpolar boreal distribution (Väinölä et al., 2008), yet its parasite assemblage has been examined in only a few studies (see the review by Bojko & Ovcharenko, 2019). Sokolov and Gordeev (2014) reported four larval helminths infecting *G. lacustris* in Kamchatka, Russia. Bojko (2017) found larval trematodes, acanthocephalans, and protozoans in populations of *G. roeselii* in Poland. Denny (1969) conducted a comprehensive study describing the metazoan parasite community of *G. lacustris* in a eutrophic Canadian lake. He found 12 species of helminths: eight cyclophyllidean cestodes, one nematode, and three acanthocephalans. The final hosts for all parasites were various bird species, as the lake harbors no permanent fish population. In the Holarctic however, *G. lacustris* is important prey for many salmonid fishes (MacNeil et al., 1999) and serves as intermediate host to several parasites that use salmonids as final hosts (Knudsen et al., 2008; Kuhn et al., 2016). In Lake Takvatn, northern Norway, *G. lacustris* is the only the amphipod in the system (Klemetsen & Elliott, 2010) and accounts for over 50% of the diet of Arctic charr (*Salvelinus alpinus*) between autumn and early winter (Prati et al., 2020), providing a key source of nutrition at a time when other prey items such as insects and zooplankton are less abundant. Hence, in subarctic lakes where fishes are common, like in Takvatn, such trophically transmitted parasites should be reflected in the assemblages of both *G. lacustris* and fish hosts.

Here, we investigate the diversity, prevalence, and abundance of parasites infecting *G. lacustris* in Lake Takvatn. Although arctic and subarctic ecosystems are generally species poor (Hoberg et al., 2012), which suggests a low diversity of parasites (Hechinger & Lafferty, 2005), recent studies show a surprisingly high taxonomic diversity of trematode parasites in Takvatn (Soldánová et al., 2017) and Icelandic lakes (Blasco‐Costa et al., 2014). Three decades of comprehensive studies of the parasite communities of snails and fishes in Takvatn indicate that *G. lacustris* should harbor parasites that use both fishes and birds as final hosts, owing to the lack of other potential final host taxa in the ecosystem (Amundsen et al., 2009, 2019; Knudsen et al., 1999). The main objectives of the present study were to: i) describe the parasite community of *G. lacustris* in a subarctic lake, and ii) describe and contrast the abundance of parasites that use fishes and birds as final hosts.

## 2 METHODS

### 2.1 Study area and collection

Takvatn (69°07′N, 19°05′E) is a subarctic, oligotrophic, and dimictic lake in northern Norway that has been the focus of intensive ecological and food web studies for more than 30 years (details in Amundsen et al., 2009, 2013, 2019). The lake is situated 214 m above sea level with a surface area of 15 km^2^ and a maximum depth of ca 80 m. There is little macrovegetation in the lake, but the littoral zone (3–10 m depth) has dense beds of the grass‐like macroalgae *Nitella* sp., which contain the highest abundances of *G. lacustris* (Frainer et al., 2016).

We sampled gammarids in the littoral zone (0–8 m depth) by dragging a benthic sled along *Nitella* sp. beds as described in Knudsen et al. (2001). Gammarids were collected from each haul, placed in buckets with lake water and vegetation, and brought back to the lab for dissection within 48 hr. Individuals not dissected within 24 hr were kept cool overnight in the refrigerator or outdoors (at approx. 4–8°C). To obtain a broader range of parasite diversity in *G. lacustris* in the lake, gammarids were collected from five sites (L1‐L5), including two in the vicinity of an important nesting area for birds (L4 and L5; Klemetsen & Knudsen, 2013). Sampling was carried out over three years during different times of the ice‐free period (August and October 2012, June and September 2013, and August 2015). Not all sites were sampled every year.

### 2.2 Dissection and parasite identification

We blotted 474 amphipods on paper towels, measured length (eye to end of telson; mm) and wet weight (g). Due to potential variation in length measurements, we generated a length–weight regression from a subsample (*y* = 132.17*x* + 5.62, *R* ^2^ = 0.95) and used weight‐based estimates of length for all analyses. We compressed whole gammarids between glass plates (150 mm × 100 mm × 3.5 mm) and examined them under a stereo microscope (Leica Wild M3, maximum magnification of 40×). Parasites were counted and transferred for further inspection under a compound microscope if needed. Parasites were identified to the nearest taxonomic level based on morphology, and select specimens were preserved in 95% ethanol for genetic analysis in a separate study (trematodes only; details in Soldánová et al., 2017) or formalin for further identification (all other parasites).

### 2.3 Statistical analyses

We characterized the parasitism in *G. lacustris* samples by calculating prevalence, mean intensity, and mean abundance (defined in Bush et al., 1997) and assessed parasite infracommunity composition using the 7‐set Venn diagram “Adelaide” (Dusa, 2020). We investigated whether the infections of parasites with indirect life cycles varied between sampling locations and sampling period, using two analyses. To analyze if the abundance of *Plagiorchis* sp. differed between sampling locations and periods, we used a mixture model (zero‐inflated negative bionomial generalized linear model (ZINB GLM); R (version 3.5.1; R Core Team, 2018), with *G. lacustris* size (length) as a covariate (Zuur et al., 2009). The ZINB GLM contains two parts; a negative binomial GLM that models parasite counts and a binomial GLM that models the probability of observing excess zeros above those of the count process (Zuur et al., 2009). Other parasite species were low in intensity so we used infection status (infected vs. uninfected) rather than abundance as the binomial response variable in logistic regressions with the same predictor variables (sampling location, period, and *G. lacustris* size).

## 3 RESULTS

### 3.1 Parasitism of *G. lacustris*

We found seven parasite taxa in 474 *G. lacustris*, where 77% (*N* = 364) of the amphipods were infected with at least one parasite (Table 1). Parasites were identified as: *Crepidostomum* spp. and *Plagiorchis* sp. (metacercariae, Trematoda, Plagiorchiida); *Cyathocephalus truncatus* (procercoid, Cestoda, Spathebothriidea); *Cystidicola farionis* (larva, Nematoda); acanthocephalan cystacanth; *Rotundula* sp. (Apicomplexa, Gregarinida); and cyclophyllidean cysticercoid (Cestoda, Cyclophyllidea, Hymenolepididae). One cysticercoid measured 325 × 275 µm (length × width), with one rostellar hook at 1 µm in length (blade = 15 µm, hook = 85 µm; rostellar hooks were not counted or removed from the cysticercoid).

**Table 1:** Parasite community of *Gammarus lacustris* in Lake Takvatn, Norway

	Final host	*N* = 474		
		Prevalence (%) (*N* hosts)	Mean abundance (Range)	*SD*
*Plagiorchis* sp.	Birds	68.6 (325)	2.68 (0–25)	3.75
Acanthocephalan cystacanth	Birds*	1.7 (8)	0.05 (0–15)	0.70
Cyclophyllidean cysticercoid	Birds*	0.4 (2)	0.26 (0–61)	3.88
*Crepidostomum* spp.	Fish	13.3 (63)	0.26 (0–8)	0.88
*Cystidicola farionis*	Fish	5.7 (27)	0.07 (0–6)	0.36
*Cyathocephalus truncatus*	Fish	2.5 (12)	0.03 (0–1)	0.16
*Rotundula* sp.^a^	N/A	12.0 (57)	1.30 (0–50)	5.00

Note

*N* = number of *G. lacustris* dissected; N hosts = N hosts infected; *SD* = standard deviation; ^a^ = *Rotundula* sp. prevalence includes only individuals with intensity data (excludes 6 individuals with only presence/absence data recorded). Asterisk indicates potential predators.

The highest prevalence was seen for the trematodes *Plagiorchis* sp. (68.6%) and *Crepidostomum* spp. (13.3%), then gregarine *Rotundula* sp. (12.0%), and the nematode *C. farionis* (5.7%). The cestodes *C. truncatus* and cyclophyllidean cysticercoids and the acanthocephalan cystacanth were rare, occurring in less than 3% of individuals (Table 1). The most abundant parasites were *Plagiorchis* sp. (2.7) and the gregarine *Rotundula* sp. (1.3). The other five species had mean abundances of less than 0.3. The cyclophyllidean cysticercoids were found in only two hosts at site L5 in 2013, at similar intensities (60 and 61).

### 3.2 Parasite community composition

**Figure 1:**
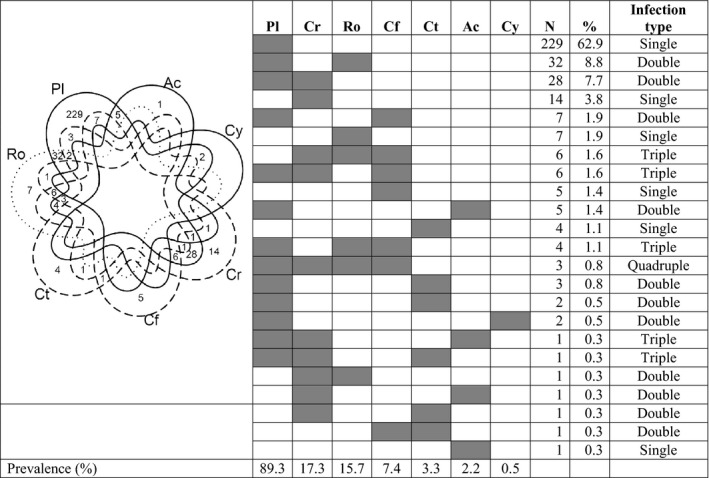
Frequency of parasite infracommunities in 364 infected *Gammarus lacustris* from Takvatn, as depicted by a 7‐set Venn diagram (“Adelaide”), where shapes of the 7 sets are identical and symmetrically rotated around the center. Data pooled across years (2012, 2013, 2015) and sites (L1‐L5). Solid lines = parasites with birds as final host; dashed lines = parasites with fishes as final hosts; dotted line = direct life cycle parasites. Shaded cells = infections, Pl = *Plagiorchis* sp., Cr = *Crepidostomum* spp., Ro = *Rotundula* sp., Cf = *Cystidicola farionis*, Ct = *Cyathocephalus truncatus*, Ac = Acanthocephalan cystacanth, and Cy = Cyclophyllidean cysticercoid. *N* = total number of hosts infected by each specific infracommunity composition. % = the percentage of hosts infected by each specific infracommunity composition

Among 364 infected *G. lacustris*, 71.4% (*n* = 260) had one parasite taxon, 22.3% (*n* = 81) had two, 5.5% had three (*n* = 20), and 0.8% (*n* = 3) had four (Figure 1). Parasites using birds as final hosts (“bird parasites”) were present in 89.3% (*n* = 325) of individuals, where *Plagiorchis* sp. was present in 99% (*n* = 323) of these hosts. Within this group, *Plagiorchis* sp. was the sole parasite species present in 62.9% (*n* = 229) of these *G. lacustris* (Figure 1). Parasites using fishes as final hosts (“fish parasites”) were present in 24.2% (*n* = 88) of infected *G. lacustris*, including 5% (*n* = 18) that had coinfections of two fish parasites. Fifteen of the 18 fish parasite coinfections consisted of the trematodes *Crepidostomum* spp. and the nematode *C. farionis*. Overall, most coinfections were with a bird and a fish parasite (*n* = 39) or a bird parasite and *Rotundula* sp. (*n* = 32). Only 2.5% (*n* = 8) of the *G. lacustris* infected with bird parasites harbored two co‐occurring bird‐parasite species. For the 23 *G. lacustris* hosts that were infected with three or four parasite species, a coinfection of both fish and bird parasites was always involved, most commonly with *Rotundula* sp. as the third species present (Figure 1). Pairwise infections were slightly more common (155) than expected (137) (chi‐square = 258, *df* = 36, *p* < .01). This was mostly due to higher than expected associations (by random assortment) between *Crepidostomum* spp. and *C. farionis* (15 observed vs. 3.6 expected) and between *C. farionis* and *Rotundula* sp. (13 observed vs. 1.4 expected).

### 3.3 Abundances of bird and fish parasites

**Figure 2:**
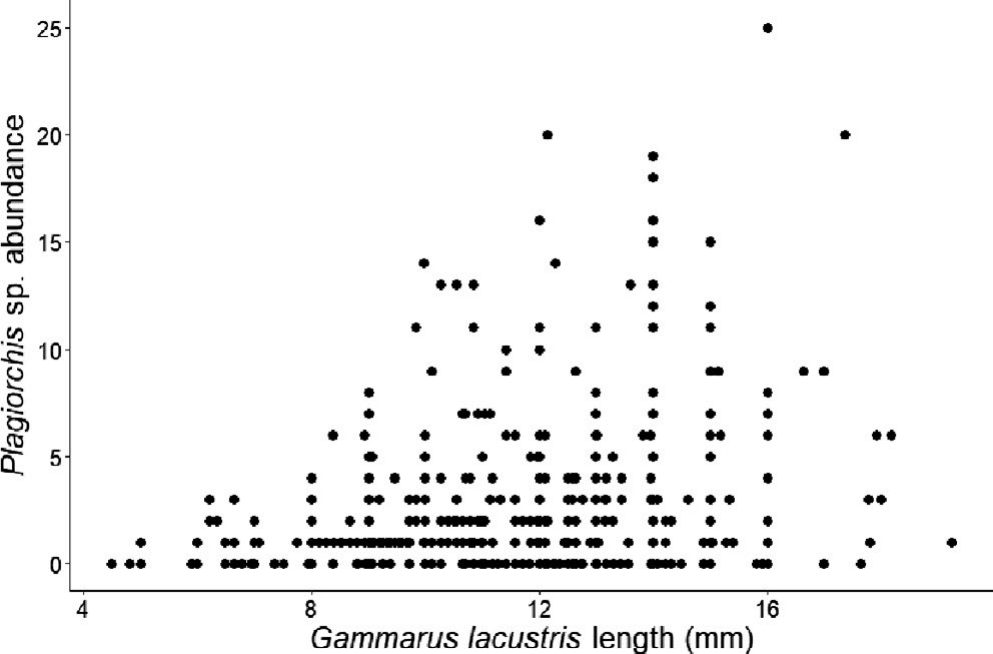
*Plagiorchis* sp. abundance plotted against *Gammarus lacustris* length (calculated from the length–weight regression)

**Figure 3:**
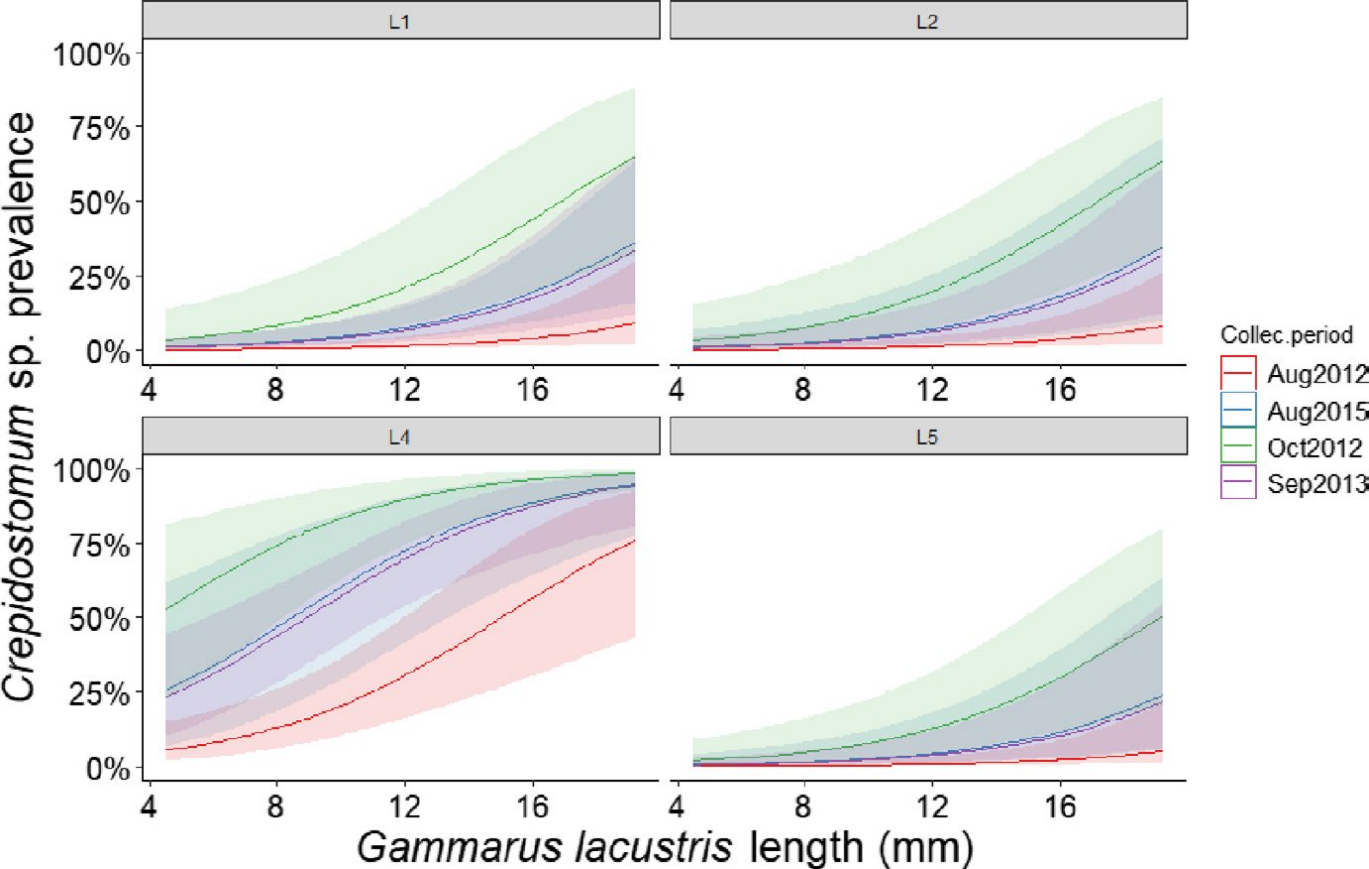
The prevalence of *Crepidostomum* spp. as predicted by the logistic regression model, by sampling site. Site L3 and June 2013 were removed due to too few observations

*Plagiorchis* sp. had the highest overall prevalence among bird parasites (Table 1). For fish parasites, *Crepidostomum* spp. were the most prevalent (Table 1). The zero‐inflated negative binomial GLM indicated that *Plagiorchis* sp. abundance increased with host size (Figure 2) and was lower in the fall season (October 2012 and September 2013) compared with late summer (August 2012 and 2015), and higher at a bird nesting site (L5; Table 2). The probability of infections with *Crepidostomum* spp. was higher at site L4 but also increased with host size and was higher in fall compared with late summer (Figure 3; Table 3). The probability of being infected with *C. farionis* also increased with host size (data not shown). For the other parasite species, however, there were no significant patterns between probability of infection and the predictors, probably due to their low prevalence in the *G. lacustris* population.

**Table 2:** Summary output from zero‐inflated negative binomial GLM predicting *Plagiorchis* sp. abundance (counts), with the estimated effects (incidence rate ratios) of predictors on *Plagiorchis* sp. counts (top) and probability of observing excess zeros (bottom). Collec. Period = collection period

Predictors	*Plagiorchis* sp. abundance		
	Incidence rate ratios	CI	*p*
(Intercept)	0.32	0.16–0.61	**0.001**
*G. lacustris* length	1.20	1.14–1.26	**<0.001**
Site [L2]	1.02	0.70–1.49	0.906
Site [L3]	1.30	0.62–2.75	0.490
Site [L4]	1.42	0.98–2.05	0.063
Site [L5]	1.99	1.40–2.83	**<0.001**
Collec. period [Aug 2015]	1.59	1.12––2.25	**0.010**
Collec. period [Jun 2013]	0.95	0.39–2.31	0.918
Collec. period [Oct 2012]	0.45	0.30–0.68	**<0.001**
Collec. period [Sep 2013]	0.49	0.36–0.65	**<0.001**
*Zero‐Inflated Model*			
(Intercept)	4,729.08	25.22–886597.29	**0.002**
*G. lacustris* length	0.26	0.12–0.57	**0.001**
Observations	462		

**Table 3:** Output from logistic regression predicting the probability of infection with *Crepidostomum* spp.

Predictors	*Crepidostomum* sp. prevalence		
	Odds ratios	CI	*p*
(Intercept)	0.00	0.00–0.00	**<.001**
*G. lacustris* length	1.31	1.15–1.51	**<.001**
Site [L2]	0.92	0.25–3.39	.901
Site [L4]	32.29	11.58–106.89	**<.001**
Site [L5]	0.55	0.12–2.16	.397
Collec. period [Aug 2015]	5.94	1.50–26.18	**.014**
Collec. period [Oct 2012]	19.48	5.98–71.07	**<.001**
Collec. period [Sep 2013]	5.25	2.10–14.48	**.001**
Observations	441		

Note

Null deviance: 364.47, *df* = 449. Residual deviance: 231.78, *df* = 441. McFadden pseudo *R* ^2^ = 0.37. Reference site and year = L1, August 2012. Site L3 and the June 2013 sampling period were removed due to too few observations.

*p*‐values are listed in bold for significant relationships.

## 4 DISCUSSION

We found seven parasite taxa infecting *G. lacustris* in Takvatn. Six of these use *G. lacustris* as an intermediate host (two trematode genera, two cestodes, one nematode, and one acanthocephalan), whereas the gregarine *Rotundula* sp. has a direct life cycle. Parasites using birds as final hosts dominated the community, with the trematode *Plagiorchis* sp. having the highest abundance and prevalence across all sampling sites and years (Table 1). With a minimum of six parasites using *G. lacustris* as an intermediate host in Takvatn, the amphipod emerges as the third most important intermediate host in Takvatn, behind first intermediate host snail *Radix balthica* (15 parasite species; Soldánová et al., 2017) and copepods (6 parasite species; Amundsen et al., 2009, 2019). Five of the 44 (11.4%) documented parasite species in Takvatn (Amundsen et al., 2009, 2013; Kuhn et al., 2015; Soldánová et al., 2017; P‐A Amundsen, unpublished data) depend exclusively on the amphipod to complete their life cycles (*Rotundula* sp., *Cystidicola farionis*, *Cyathocephalus truncatus*, acanthocephalan cystacanth, and the cyclophyllidean cysticercoid) (Table 4). Thirty‐two of the 44 documented parasite species in Takvatn are helminths, and six (16%) of these helminths use or require *G. lacustris* in their life cycles. All of these parasites are then able to infect a range of predators as final hosts—three fish species, seven bird species (Table 4). In light of the high parasite diversity found in and dependent on *G. lacustris*, the amphipod emerges as a key species for parasite transmission in Takvatn, and likely in other subarctic lakes where it often occurs.

**Table 4:** Parasite and predator species dependent on *Gammarus lacustris* in the Takvatn food web

Parasite (life stage, taxa)	Predator
*Rotundula* sp. (Apicomplexa, Gregarinida)	N/A (direct life cycle)
*Cystidicola farionis* (larva, Nematoda)	*Salvelinus alpinus*
Acanthocephlan cystacanth (Acanthocephala)	*Gavia arctica* (Black‐throated loon)*
	*Anas* spp. (Dabbling ducks)*
	*Aythya fuligula* (Tufted duck)*
	*Melanitta nigra* (Common scoter)*
	*Melanitta fusca* (Velvet scoter)
	*Bucephala clangula* (Common goldeneye)*
	*Mergus serrator* (Red‐breasted merganser)*
*Cyathocephalus truncatus* (procercoid, Cestoda, Spathebothriidea)	*Gasterosteus aculeatus* (Three‐spined stickleback)
	*S. alpinus* (Arctic charr)
	*Salmo trutta* (Brown trout)
Cyclophyllidean cysticercoid (Cestoda, Cyclophyllidea, Hymenolepididae)	A. fuligula*
	M. nigra*
	M. fusca*
	B. clangula*
	M. serrator*

Note

Asterisk indicates potential predators.

Actual parasite diversity in Takvatn *G. lacustris* could be higher than seven species. Although the sampling of 474 amphipods likely captured most common parasite species, finding two rare (<10 infected hosts) parasite species indicated that our sampling effort might have missed other rare parasite species present at Takvatn. Therefore, to compare the total parasite richness estimate for *G. lacustris* in Takvatn with other sites, the estimate of 7 species has 95% confidence limits between 6 and 8 species (Hsieh et al., 2016). Further, some “species” might represent species complexes, due to the potential for cryptic species—an increasingly common discovery among helminth parasites, especially trematodes (Gordy & Hanington, 2019). Soldánová et al. (2017) recently examined trematode diversity in *R. balthica*, *G. lacustris*, and other invertebrates in Takvatn, using samples that were collected during the present study, and revealed four genetically different species of *Crepidostomum*: *C. metoecus*, *C. farionis*, plus two new cryptic species (*Crepidostomum* sp. 1 and 2 in Soldánová et al. (2017)). Of the four genetically distinct *Crepidostomum* spp., only *C. metoecus* is confirmed as infecting *G. lacustris* in Takvatn (Soldánová et al., 2017). However, *Crepidostomum farionis* is reported in *Gammarus pulex* from other northern systems (Awachie, 1968) and is likely able to infect *G. lacustris* in Takvatn, as are the two other species (M. Soldánová, personal communication). Therefore, we decided to use “*Crepidostomum* spp.” in the present paper rather than limit the identification to *C. metoecus*. Additionally, Soldánová et al. (2017) molecularly identified one species of *Plagiorchis* from *G. lacustris* (*Plagiorchis* sp. 2) but report seven genetic lineages of *Plagiorchis* in total—most with unknown life cycles. We observed *Plagiorchis* sp. metacercariae in numerous other invertebrates sampled at Takvatn; hence, “*Plagiorchis* sp.” here potentially represents multiple cryptic species. Finally, the gregarine *Rotundula* sp. can co‐occur with other gregarines in amphipods, (as reported by Grunberg & Sukhdeo, 2017). Altogether, these studies indicate that our results likely underestimate the number of parasite species in *G. lacustris* in Takvatn. More definitive identification of the parasites could be obtained by sampling adult helminths from final bird and fish hosts and matching their DNA to the larval stages that we identified; however, this additional sampling was beyond the scope of the present study.

### 4.1 Bird parasites

The persistently high abundance and prevalence of *Plagiorchis* sp. (Table 1) are likely related to the relatively rich and stable aquatic bird community (Klemetsen & Knudsen, 2013), the putative final hosts for *Plagiorchis* sp. in Takvatn, and also the high abundance of its first intermediate snail host *R. balthica* (Klemetsen & Elliott, 2010), as final host diversity and abundance can drive abundance and diversity in larval trematode communities in intermediate host snails (Hechinger & Lafferty, 2005). The highest abundance of *Plagiorchis* sp. in *G. lacustris* occurred near the bird nesting islets in August. Although the genus *Plagiorchis* infects a broad range of vertebrate final hosts, including amphibians, reptiles, birds, and mammals, birds are the putative final hosts for *Plagiorchis* sp. in Takvatn, due to the absence of other potential final host taxa (Amundsen et al., 2009, 2019; Knudsen et al., 1999).

The acanthocephalan cystacanths were likely *Polymorphus* sp., based on morphological identification and published records in the region (Tomáš Scholz, Czech Academy of Sciences, personal communication). Prevalence in *G. lacustris* remained low across sites and years, which is in contrast to the Canadian study reporting 12.7% prevalence for *Polymorphus marilis* (Denny, 1969). However, since many acanthocephalan parasites alter the behavior of their *Gammarus* host by increasing their vulnerability to predation by bird final hosts (Bakker et al., 2017; Helluy & Thomas, 2010; Jacquin et al., 2014; Lagrue et al., 2013), their ecological significance can be high despite a low prevalence. The potential for behavior modification in the system may also mean that the low prevalence we observed could result from the preferential selection of infected amphipods by nonhost fish or bird predators (Knudsen et al., 2001; Lafferty, 1999; Song & Proctor, 2020; Ubeda et al., 1994).

The cyclophyllidean cysticercoid occurred in only two of the 474 *G. lacustris* specimens. Cyclophyllidean cysticercoids have been reported from gammarid amphipods, including *Lateriporus teres* and *Microsomacanthus microsoma* in *G. lacustris* (Nikolov et al., 2008), *Microsomacanthus pachycephala* in *Echinogammarus stammeri* (Dezfuli et al., 2002), and two species of unidentified cysticercoids in *Hyallela patagonica* (Rauque & Semenas, 2013). The size and shape of the Takvatn cysticercoids and their rostellar hooks resemble *M. microsoma* more than *L*. *teres*, but their species identity has not been established. The two gammarid specimens containing cysticeroids were both collected at a site with high bird abundances, which are the final hosts for most hymenolepidid cestodes, including those previously reported in gammarid amphipods (Dezfuli et al., 2002; Nikolov et al., 2008).

### 4.2 Fish parasites

*Crepidostomum* spp. were the most common of the three fish parasite species (Table 1) and more prevalent at Takvatn than the 2% prevalence observed in *G. lacustris* from another subarctic lake (Sokolov & Gordeev, 2014). Adult *Crepidostomum* spp. frequently infect Arctic charr in Takvatn (Kuhn et al., 2016) and are common in other lakes with salmonids (Arctic charr and brown trout, *Salmo trutta*) in the region (Knudsen, 1995; Knudsen et al., 2008; Siwertsson et al., 2016). *Crepidostomum* spp. infect other vertebrates as final hosts, including amphibians and reptiles, but fish are the putative final hosts in Takvatn, as the other potential final host taxa are absent (Amundsen et al., 2009, 2019; Knudsen et al., 1999). Interestingly, 38% of the *Crepidostomum* spp. metacercariae were progenetic. Progenesis would be an advantageous strategy for *Crepidostomum* spp. if predation by appropriate final hosts (fishes) was uncommon (as might occur at lakes without fishes). The pattern of *Crepidostomum* spp. progenesis at Takvatn has yet to be explored, but Lagrue and Poulin (2009) report that *Coitocaecum parvum*, another allocreadiid trematode, becomes progenetic in the absence of chemical cues from the definitive host fish.

Prevalence of the nematode *C. farionis* was slightly higher than the 1%–4% reported previously (Knudsen et al., 1999). Other studies have observed equal (Awachie, 1973) or higher (Sokolov & Gordeev, 2014) prevalences of *C. farionis* in *G. lacustris*. However, in the nearby lake, Fjellfrøsvatn, within the same drainage as Takvatn, *C. farionis* is rare in *G. lacustris* (0.2%; Knudsen et al., 2001). Despite the relatively low prevalence in *G. lacustris* in both lakes, *C. farionis* is prevalent in Arctic charr in Takvatn and Fjellfrøsvatn (Knudsen et al., 2002, 2004) perhaps because these worms are long‐lived and accumulate over time in fish hosts (Moravec, 1994). Higher than expected double infections observed between *C. farionis* and *Crepidostomum* spp., and *C. farionis* and *Rotundula* sp. might indicate overlapping infection risk among parasite species in space, habitat or host demographics, or that *C. farionis* might alter host susceptibility, increasing the risk of coinfections.

The cestode larva *Cyathocephalus truncatus* had a higher prevalence in *G. lacustris* in Takvatn (Table 1) than in nearby Fjellfrøsvatn (0.6%). In Fjellfrøsvatn, the infection increased with host size, reaching nearly 4% in large *G. lacustris* (Knudsen et al., 2001), a pattern not revealed in the present study. Other studies of *C. truncatus* in *Gammarus* spp. report similar prevalence levels as in Takvatn, such as from Kamchatka, Russia (2.9%; Sokolov & Gordeev, 2014), although very low prevalences have been observed in other amphipod hosts (see Awachie, 1966; Dezfuli et al., 2000). Despite the moderate infection levels of *C. truncatus* in *G. lacustris*, the infection in the final salmonid hosts in this region can be very high (50%–70%; Amundsen et al., 2003), which has been shown to be a result of selective predation on infected amphipods (Franceschi et al., 2007; Knudsen et al., 2001).

### 4.3 Direct life cycle parasites

The prevalence of the gregarine *Rotundula* sp. ranged from 2% to 28% among sites and years (Supplementary Table S2), which is lower than the 40%–90% found in other studies of *Gammarus* spp. (Bojko et al., 2017; Grunberg & Sukhdeo, 2017; Sorcetti & Di Giovanni, 1984). Even though intensities of gregarines can be high (>50), as seen in Takvatn, the pathology of these infections may be limited (Bojko et al., 2017; Grunberg & Sukhdeo, 2017). Our total lengths for *Rotundula* sp. were greater than those previously reported from *Gammarus* spp. (Goodrich, 1949; Sorcetti & Di Giovanni, 1984). Possible explanations for the variability in length could be that *Rotundula* sp. at Takvatn is a different species than those previously described, or the small sample sizes in the published studies do not represent the full range of sizes found in those populations from immature trophs to mature gamonts (Tamara Cook, Sam Houston State University, personal communication); our study certainly did not comprehensively sample enough individuals to obtain a full picture of the *Rotundula* sp. population at Takvatn.

## 5 CONCLUSION

We found a high parasite diversity and high prevalences of several parasite taxa in *G. lacustris*, including parasites that use birds and fishes as final hosts. In fact, 16% of the total helminth diversity in Takvatn is dependent on the amphipod. Some of these parasites likely exert an impact on the functional role of *G. lacustris* in the ecosystem (Frainer et al., 2018), either by reducing host‐foraging performance, or through behavioral manipulations that increase its predation susceptibility. Thus, parasites exert both top‐down and bottom‐up effects in the Takvatn trophic network. *Gammarus lacustris* has dual and substantial roles in the lacustrine food web—it is an important food source for fishes and birds and maintains parasite diversity as an obligate second intermediate host for several parasite species.

## 6 DATA ACCESSIBILITY STATEMENT

All data supporting this study can be accessed from Dryad: https://doi.org/10.25349/D9B89T.

## CONFLICT OF INTEREST

The authors declare that they have no competing interests.

## AUTHOR CONTRIBUTION

**Jenny C Shaw:** Data curation (equal); Formal analysis (equal); Investigation (equal); Writing‐original draft (equal); Writing‐review & editing (equal). **Eirik Haugstvedt Henriksen:** Data curation (equal); Formal analysis (equal); Investigation (equal); Writing‐original draft (equal); Writing‐review & editing (equal). **Rune Knudsen:** Conceptualization (equal); Investigation (equal); Writing‐review & editing (equal). **Jesper Kuhn:** Data curation (equal); Formal analysis (equal); Investigation (equal). **Armand M Kuris:** Conceptualization (equal); Investigation (equal); Writing‐review & editing (equal). **Kevin D Lafferty:** Conceptualization (equal); Formal analysis (equal); Investigation (equal); Writing‐review & editing (equal). **Anna Siwertsson:** Data curation (equal); Investigation (equal). **Miroslava Soldanova:** Investigation (equal); Writing‐review & editing (equal). **Per‐Arne Amundsen:** Conceptualization (equal); Funding acquisition (equal); Investigation (equal); Writing‐review & editing (equal).
